# High resolution functional analysis and community structure of photogranules

**DOI:** 10.1038/s41396-023-01394-0

**Published:** 2023-03-30

**Authors:** Lukas M. Trebuch, Olivia M. Bourceau, Stijn M. F. Vaessen, Thomas R. Neu, Marcel Janssen, Dirk de Beer, Louise E. M. Vet, René H. Wijffels, Tânia V. Fernandes

**Affiliations:** 1 Department of Aquatic Ecology, Netherlands Institute of Ecology (NIOO-KNAW), Droevendaalsesteeg 10, 6708 PB Wageningen, The Netherlands; 2 Bioprocess Engineering, AlgaePARC Wageningen University, P.O. Box 16, 6700 AA Wageningen, The Netherlands; 3 Microsensor Research Group, Max-Plank-Institute for Marine Microbiology, Celsiusstrasse 1, 28359 Bremen, Germany; 4 Microbiology of Interfaces, Department River Ecology, Helmholtz Centre for Environmental Research - UFZ, Brueckstrasse 3A, 39114 Magdeburg, Germany; 5 Department of Terrestrial Ecology, Netherlands Institute of Ecology (NIOO-KNAW), Droevendaalsesteeg 10, 6708 PB Wageningen, The Netherlands; 6 Faculty of Biosciences and Aquaculture, Nord University, N-8049 Bodø, Norway

**Keywords:** Biofilms, Microbial ecology

## Abstract

Photogranules are spherical aggregates formed of complex phototrophic ecosystems with potential for “aeration-free” wastewater treatment. Photogranules from a sequencing batch reactor were investigated by fluorescence microscopy, 16S/18S rRNA gene amplicon sequencing, microsensors, and stable- and radioisotope incubations to determine the granules’ composition, nutrient distribution, and light, carbon, and nitrogen budgets. The photogranules were biologically and chemically stratified, with filamentous cyanobacteria arranged in discrete layers and forming a scaffold to which other organisms were attached. Oxygen, nitrate, and light gradients were also detectable. Photosynthetic activity and nitrification were both predominantly restricted to the outer 500 µm, but while photosynthesis was relatively insensitive to the oxygen and nutrient (ammonium, phosphate, acetate) concentrations tested, nitrification was highly sensitive. Oxygen was cycled internally, with oxygen produced through photosynthesis rapidly consumed by aerobic respiration and nitrification. Oxygen production and consumption were well balanced. Similarly, nitrogen was cycled through paired nitrification and denitrification, and carbon was exchanged through photosynthesis and respiration. Our findings highlight that photogranules are complete, complex ecosystems with multiple linked nutrient cycles and will aid engineering decisions in photogranular wastewater treatment.

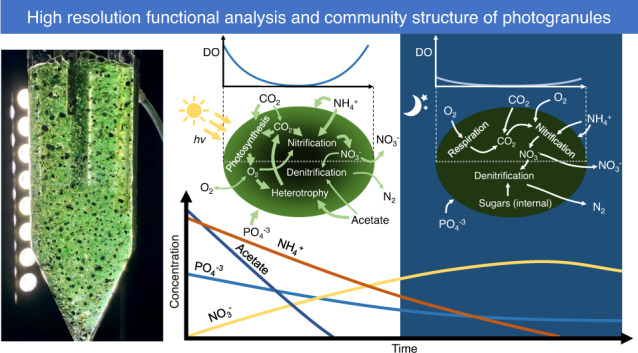

## Introduction

Wastewater treatment reactors continuously select for functional traits in their microbial communities. Manipulating operating conditions allows for the selection of desired traits, including conversion processes to purify water and self-aggregation of microbial biomass. Self-aggregation (i.e., forming biogranules, spherical aggregates of microorganisms) enables stratification (oxic and anoxic zones) and facilitates biomass harvesting because dense aggregates rapidly sink once reactor mixing is stopped. Generally, biogranules are formed in reactors where the liquid residence time is shorter than the doubling time of the microorganisms. This washes out suspended cells and generates selective pressure for biomass retention [1, 2]. Although the aggregated biomass in biogranules is subject to mass transfer resistances that reduce the activity per cell, efficient biomass retention and the ensuing increased biomass assures strongly elevated volumetric conversion rates in biogranule reactors.

Phototrophic biogranules, called photogranules, were first observed in cultures of photosynthetic mats from the North Sea [3]. The granules were composed of filamentous cyanobacteria, diatoms, and heterotrophic bacteria. Spherical geometry is rare in phototrophic communities, but some examples of photogranules in nature have been reported. For example, photogranules composed of cyanobacteria and heterotrophic bacteria called cryoconites are found in glaciers [4, 5]. Green and pink microbial “berries” are found in salt marshes, formed through a symbiosis of cyanobacteria and diatoms, and of communities of sulfur-oxidizing purple sulfur bacteria and sulfur-reducing bacteria, respectively [6, 7].

In phototrophic biofilms in nature, as in photogranules, concentration gradients of various dissolved chemical species (e.g., oxygen, substrates) are formed due to diffusional limitation. Similarly, light intensity gradients are formed by light absorption and scattering [8]. These intersecting gradients create a varied environment that can support the simultaneous growth of diverse microorganisms filling different niches, such as photoautotrophs, chemoautotrophs, and heterotrophs, exhibiting aerobic, and anaerobic metabolisms [9]. Complex interactions between these microbial groups take place, that may stabilize the functioning of the consortium [10]. For example, heterotrophs may grow on extracellular organic compounds excreted by phototrophs. The latter, in turn, may fix the inorganic CO_2_ produced in heterotrophic growth. Aerobic chemoautotrophs, such as nitrifiers, may benefit from photosynthesis-enhanced oxygen levels while simultaneously competing with phototrophs for inorganic carbon and nitrogen. Unlike in biofilms, photogranules are free living, so their biomass is not limited to the surface area of their container. Furthermore, the surface to volume ratio of a sphere is such that for small spheres, there is capacity for a higher biomass-water exchange than in a plane of the same volume. This relationship holds so long as the radius of the sphere is less than one third of the thickness of the plane.

Recently, photogranules were cultivated with selection pressure to remove and recover nitrogen, phosphorus, and carbon from wastewater [11–17]. This involved a regular exchange of the medium after settling of biomass, thus with a selection pressure towards formation of fast-settling granules. The granules exhibited indeed excellent settling properties, and their in-situ photosynthetic oxygen production fuelled oxygen-demanding microbial processes such as nitrification and respiration. This linked the O_2,_ and CO_2_ cycles within the treatment process, making progress towards “aeration-free” wastewater treatment. Initial studies have investigated the physical structure and metabolic functions of single photogranules from wastewater, but were limited in focus to phototrophic organisms and oxygen profiles [18–20]. A modelling approach predicted the distribution of microorganisms and extracellular polymeric substances (EPS) within photogranules and the bulk turnover of chemical compounds and reactor functions based on varying nutrient inputs, but has not yet been tested in real photogranules [21, 22].

Photogranules experience both varying external environmental conditions (i.e., light intensity, nutrient concentrations) during reactor operation, and internal conditions, as nutrient gradients are created and eliminated in response to microbial activity and external variation. Therefore, a full understanding of the microbial ecology within photogranules requires detailed investigation under various and varying conditions in vivo. Here, we studied the physical and biological stratification and functioning of these same photogranules with microscopic imaging, metataxonomics, microsensors, and incubations with radio- and stable-isotope labels. Our findings provide insight into the spatial and temporal distribution of functional activity (photosynthesis, nitrification, and denitrification) within photogranules and their dependency on external factors (light, nutrients). Further, the results can be used to support engineering decisions in photogranular wastewater treatment.

## Materials and methods

### Cultivation and sample preparation

Photogranules were obtained from bioreactors as described previously [12] (Fig. S1). The bioreactors were bubble columns 38 cm high, 10 cm in diameter and with a bottom cone 10 cm high. The working volume was 1.6 L, standing 28 cm from the bottom of the cone. Mixing was achieved by gassing with air enriched with 5% v/v CO_2_ at a rate of 500 mL min^−1^. Temperature was kept at 35 °C using an external water bath and pH at 6.8 ± 0.1 by automatic addition of 1 M HCl or 1 M NaOH. Bioreactors were operated in sequencing batch mode with a settling time of 5 min, a hydraulic retention time (HRT) of 0.67 days, and an operating cycle of 12 h. Day:night cycles of 12 h were superimposed on the sequencing batch cycle such that each batch cycle was subjected to 6 h light and 6 h darkness. Warm white light (4000 K) LED lamps (Avago ASMT-MY22-NMP00, Broadcom Inc., USA), positioned at one side of the bioreactor, were providing an incident light intensity of 500 µmol m^−2^ s^−1^ at the surface of the bioreactor during the light phase. A sludge retention time (SRT) of 7 days was achieved by automatically removing 114 ml of the mixed liquor at the end of every batch cycle via a peristaltic pump. The influent contained 100 mg_N_ L^−1^ (7.1 mmol_N_ L^−1^) as ammonium, 10 mg_P_ L^−1^ (0.3 mmol_P_ L^−1^) and 200 mg_COD_ L^−1^ (3.2 mmol L^−1^ of sodium acetate). The bioreactors were operated for 299 days. Photogranules were sampled during stable operational periods at day 105, 186, 214, 270, and 299 from the mixed phase of the bioreactor and were either directly analyzed or fixed in paraformaldehyde (PFA) in 5% phosphate buffered saline (PBS) for later microscopic analysis. Photogranules ranged from 0.4 to 5 mm wide with an average diameter of 2.6 mm. Photogranules ranging from 2 to 4 mm wide were used for analysis.

### White light microscopy and confocal laser scanning microscopy (CLSM)

A stereomicroscope (Leica M205C, Germany) was used to visualize whole and sectioned photogranules under white light. Images were obtained with the Leica Application Suite (LAS version 4.13, Germany). The 3-dimensional structure of the granules was examined by multi-channel CLSM (Leica TCS SP5X, Germany). The system with an upright microscope and a super continuum light source was controlled by the LAS-AF 2.4.1 software. Image data stacks were recorded with 25× NA 0.95 and 63× NA 1.2 water immersion lenses. Samples were mounted in a cover well chamber with spacers. For this purpose, the photogranules were cut in half and stained inside the chamber, which was then filled up with water and closed with a coverslip. For identifying a suitable lectin, a screening with all commercially available lectins was performed. Glycoconjugates were detected by fluorescence lectin-binding analysis (FLBA), according to Staudt et al. and Zippel and Neu [22, 23]. After testing several lectins, the BAN lectin labelled with Alexa-568 was selected for imaging. BAN is a lectin derived from banana (*Musa paradisiaca*), which has single-carbohydrate binding specificity for d-mannose and d-glucose [24]. The fluorochrome Syto9 was used as a counterstain to visualize nucleic acids. Cyanobacteria and eukaryotic algae were identified based on their pigments [25, 26]. The settings for recording image data stacks sequentially were as follows: Excitation: 480, 635 nm and 565 nm, emission: 500–550 nm (Syto9), 585–650 nm (BAN-A568, phycobilins), 650–720 nm (Chl A). Images were processed with the imaging software Fiji [27] and individual image data stacks were stitched together using Photoshop (version CS6).

### 16S/18S rRNA gene amplicon sequencing

DNA samples were taken to assess the microbial community. Specifically, 15 mL of harvested photogranules were homogenized by a glass/Teflon tissue grinder, aliquoted into five 2 mL microcentrifuge tubes, centrifuged at 14.87 × 10^3^ rcf for 10 min and the supernatant discarded. The cell pellets were immediately frozen at −80 °C until further processing. DNA of 200 mg of wet cell pellet was extracted in triplicate by using the DNeasy PowerSoil Pro Isolation Kit (Qiagen GmbH, Hilden, Germany) according to the manufacturer’s protocol. The quantity and quality of DNA were spectrophotometrically determined with a NanoDrop One (ThermoFisher Scientific, USA). The DNA samples were submitted for sequencing to Génome Québec (McGill University, Montreal, CA). The 16S rRNA gene V3/V4 variable region was amplified using primer pair 341F (CCTACGGGNGGCWGCAG) and 805R (GACTACHVGGGTATCTAATCC) [28]. The 18S rRNA gene V4 variable region was amplified using the primer pair 616*F (TTAAARVGYTCGTAGTYG) and 1132R (CCGTCAATTHCTTYAART) [29]. Both sets of primers were modified to add Illumina adaptor overhang nucleotides sequences to the gene-specific sequences. Sequencing was performed using a MiSeq system (Illumina, USA) with 300-bp reads (v3 chemistry). Primers were removed from the raw sequences using *cutadapt* (version 1.18) [30]. The obtained sequences were processed further with DADA2 programme [31]. Taxonomic alignment of the sequences was done to the SILVA database (release 138) using SINA (https://www.arb-silva.de). The 16S and 18S dataset was normalized using the cumulative sums scaling (CSS) function of the R package *metagenomSeq* version 1.24.1 [32]. The analysis of the microbiome data was performed with the R-package *phyloseq* (version 1.26.1) [33]. The raw 16S and 18S rRNA gene sequence data is available in the EBI database under project number PRJEB54633.

### Microsensor measurements

Photogranules were pinned with glass needles to a nylon mesh fitted over a small petri dish (Fig. S2). The Petri dish was submerged in tap water (Table S1) and amended with acetate, medium stocks, and nitrate as indicated. Oxygen concentration was determined using a Clark-type oxygen microsensor [34] fitted to a motorized micromanipulator and two-point calibrated in oxygen-saturated water and basic sodium ascorbate (oxygen-free baseline). Nitrate concentrations were determined using a nitrate LIX membrane sensor manufactured in house (Max Planck Institute for Marine Microbiology, Bremen, Germany), calibrated in a nitrate dilution series [35]. Light profiles were determined with a scalar irradiance light microsensor with an 80 µm spherical tip (Zensor, Denmark) [36], connected to a USB4000 fibre optic spectrophotometer (Ocean Optics, USA) [37]. To collect profiles, microsensors pierced the granule, propelled stepwise by a motorized micromanipulator.

Using the oxygen and nitrate profiles generated from the microsensor measurements, the net consumption/production rates of reactants were calculated by segregating the granule into “shells,” with each shell the width of the distance between measurements, and assuming a spherical geometry. The flux between a point on the outer part of the shell and a point on the inner part of the shell was calculated, then multiplied by the surface area of the shell.

1 $$P = D \times \frac{{dC_r}}{{dx_r}} \times 4 \times {{{{{\rm{pi}}}}}} \times r^2$$

with *P* as the conversion rate per shell of aggregate (expressed in this paper in nmol/h), with *D* as diffusion coefficient, *dC*_*r*_*/dx*_*r*_ the concentration gradient of the reactant at location *r*, and *r* the radial distance from the shell surface to the centre of the aggregate. The total conversion rates were obtained by addition of activities in all shells, since the rates represent total activities, not activities normalized to volume. To produce the volumetric rate the production rate of each shell was divided by the volume of that shell. The diffusion coefficient used for oxygen was 2000 µm^2^ s^−1^, (Bionumbers ID 104440) [38, 39] and the coefficient used for nitrate was 1700 µm^2^ s^−1^ (Bionumbers ID 104439) [38, 39].

Separate photosynthesis rates were determined by placing the microsensor at a set depth, then covering the light for 5–10 s. The rate of the decrease in oxygen concentration is equivalent to the rate of photosynthesis at that depth.

### ^14^C carbon fixation incubations

Photogranules were incubated without headspace in 5.9 mL glass vials at pH 6.5 in tap water amended with either 3 mM or 10 mM sodium bicarbonate, and amended with 8 mM ammonium, 3 mM phosphate, and/or 3 mM acetate as indicated. There were 3–4 photogranules per vial, with one vial per condition, except for the incubation with ammonium and phosphate, which had 2 vials. Additionally, photogranules were provided with ~60 kBq ^14^C bicarbonate. Vials were constantly rotated and incubated for 6 h at room temperature in the dark (nitrification experiment) or in the light (photosynthesis, ~250 µmol m^−2^ s^−1^). Incubations were performed simultaneously. Incubations were stopped by removing 600 µL supernatant and replacing it with a 20% paraformaldehyde (PFA) solution,  achieving a final concentration of 2% PFA. Photogranules used for control (blanks) were killed first in PFA and then exposed to the tracer for 6 h.

The photogranules were washed twice in 10 mM carbonate buffer to remove unreacted carbonate tracer and embedded by immersing in optimal cutting temperature compound (OCT, Leica) overnight, then frozen in 1 ml plastic cups at −20 °C and sectioned in 20 µm thick slices in a cryomicrotome. The slices were caught on polylysine slides, and the radioactivity distribution was imaged in a radio-imager (BioSpaceLab, Paris), until 10^6^ counts were obtained. Blanks were counted for 12 h, as 10^6^ counts could not be obtained in a reasonable time. The produced images were then analyzed with M3Vision (BioSpaceLab, Paris). The CO_2_ uptake of the whole granule was obtained, subsequently normalized per mm^2^ and then divided by the thickness of the aggregate section to obtain volumetric rates (see Fig. S6).

### ^15^N nitrogen incubations

Photogranules were incubated in 6 mL gas-tight glass vials in tap water amended with 3 mM sodium bicarbonate and either 1 mM ^15^N ammonium or 1 mM ^15^N nitrate and incubated without headspace at room temperature in the dark or light as indicated. Two photogranules were placed in each vial, one small and one large. Four separate vials were prepared per condition, and the photogranules in one vial per group were killed at 4 separate time points (~0, 2, 4.5, and 5.5 h incubation). Photogranules were killed with a 1:1 w/v ZnCl solution. Timepoint 0 was used as the blank.

After killing the photogranule, a 2 mL helium headspace was created in each vial. The vials were shaken vigorously and allowed to equilibrate for 5 days. Subsequently, 150 µL of headspace was injected into an IR-MS and analyzed for ^15^N-N_2_. Injections were calibrated against a series of ambient air injections, allowing calculation of the excess ^15^N nitrogen content. Total excess ^15^N was calculated as (excess ^29^N_2_ + (2 × excess ^30^N_2_)). Rates of denitrification were calculated by fitting a linear equation to the excess ^15^N production [40].

NO_x_, the sum of nitrate and nitrite, was converted to NO with acidic vanadium chloride and measured with a CLD 60 Chemiluminescence NO/NO_x_ analyser [41]. NO_x_ content was calibrated against a dilution series of nitrate. The rate of nitrification was then determined by fitting a linear equation to the sum of the excess ^15^N at each time point and the NO_x_ concentration.

## Results

### Physical structure of photogranules

**Fig. 1:**
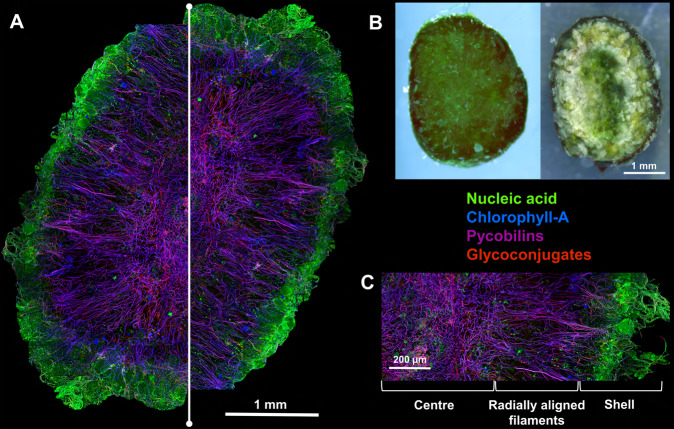
Macro-photographs and CLSM of photogranules. **A** CLSM image of a photogranule cross section showing the nucleic acid staining (green), photopigments of filamentous cyanobacteria as an overlay of phycobilins (red) and chlorophyll A (blue) resulting in purple, the chlorophyll A of eukaryotic microalgae (blue), and the lectin signal of glycoconjugates (red). The CLSM images are made of 14 individual images stitched together. Due to limitations of the CLSM technique only half of the photogranule could be captured and for representation purposes the image was mirrored over the white vertical line. **B** Macro-photography of a photogranule under white light. The left image shows the whole photogranule and the right image the cross section of the same photogranule. **C** Close-up of the CLSM cross section from the centre to the surface of the photogranule. The cross section can be divided into three distinct zones: (1) the centre, (2) radially aligned filaments and (3) the shell.

The photogranules were physically robust and exhibited a clear stratification (Fig. 1). Filamentous cyanobacteria (phycobilin and Chl A autofluorescence) with gliding motility formed a complex network that functioned as a scaffold for other microorganisms. A dense shell of both phototrophic and non-phototrophic organisms (stained by SYTO9) formed the outer 300–500 µm of the granule. Below this shell was a zone of radially aligned filamentous cyanobacteria followed by a dense and jumbled centre. Coccoid eukaryotic algae (Chl-a autofluorescence) were present in microcolonies throughout the photogranule (Fig. S4). Glycoconjugates (visualized by lectin staining) surrounded the filamentous cyanobacteria throughout the entire photogranule (Fig. S4D). Glycoconjugates indicate the excretion of extracellular polymeric substances (EPS) by phototrophic and non-phototrophic organisms and can contribute to the physical structure of the photogranule, serving as a “glue.” The BAN lectin used in this study visualizes glycoconjugates with d-glucose and d-mannose monosaccharide building blocks.

### Microbial community composition

**Fig. 2:**
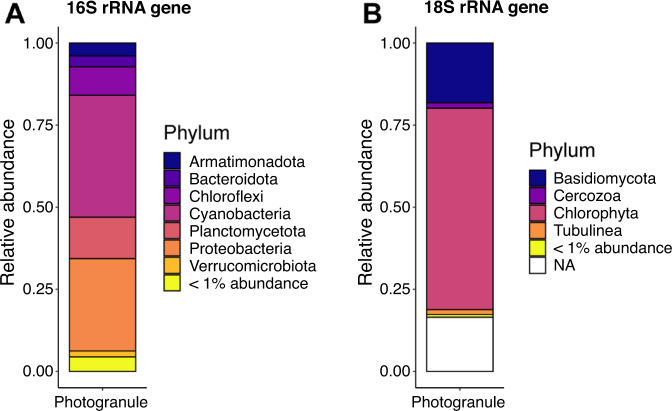
Microbial community composition obtained by 16S/18S rRNA gene amplicon sequencing. The relative abundance is given at the Phylum level. All ASVs with abundances lower than 1% are grouped together and displayed as “< 1% abundance” in the two bar plots.“NA” are ASVs that are not assigned at phylum level. **A** 16S dataset and **B** 18S dataset.

The microbial community consisted of both phototrophic organisms, including motile filamentous cyanobacteria and eukaryotic algae, and non-phototrophic organisms (Fig. 2A). The 16S rRNA gene sequences were dominated by amplicon sequencing variants (ASV) attributed to phototrophic organisms from the phylum *Cyanobacteria* (37%) and non-phototrophic organisms from the class *Proteabacteria* (28%). The two most abundant ASV were the cyanobacteria *Leptolyngbya boryana* with 15% relative abundance and *Alkalinema pantanalense* with 13% relative abundance. The nitrifiers *Nitrosomonas* sp., *Nitrobacter* sp. and *Nitrospira* sp. made up about 2% of the prokaryotic community, while the aerobic chemoheterotrophs and denitrifiers *Thauera* sp. and *Zoogloea* sp. made up 15% relative abundance. Strict anaerobic prokaryotes from the Family *Anaerolineaceae* and *Caldilineaceae* together made up 5% of ASVs, suggesting that part of the photogranule was anoxic. This is indeed confirmed by microsensing in the following section. The 18S rRNA gene sequences were dominated by ASVs attributed to eukaryotic microalgae (58%), fungi (18%), and protists (2%) (Fig. 2B). The eukaryotic algae present were *Chlorella* sp. (39%), *Chlorococcum* sp. (13%), *Botryoshaerella* sp. (1%), and *Tetradesmus* sp. (1%). The fungi present was *Trichosporon* sp. (18%).

### Light absorption

**Fig. 3:**
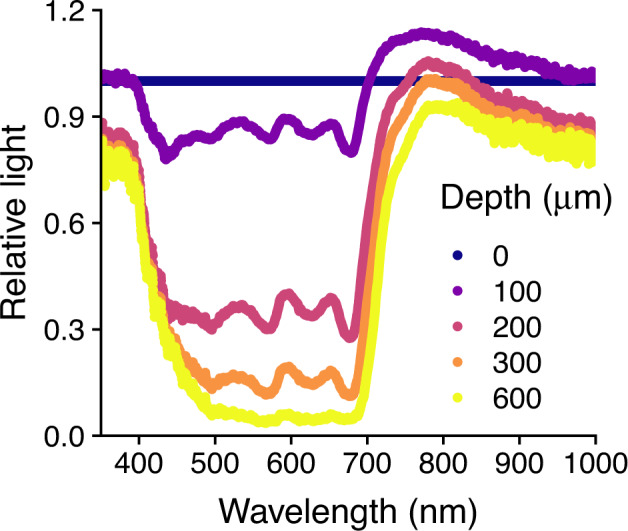
The light relative to the photogranule surface, measured with a light microsensor, is plotted for five different depths. Surface illumination was approximately 250 µmol m^-2^ s^−1^.

The photogranules effectively absorbed light across the visible light spectrum (Fig. 3). Within 600 µm depth 90% of the surface light was completely absorbed. There was an increase in scalar irradiance in the range of 700–800 nm especially at 100 and 200 µm depth when compared to the reference light intensity at 0 µm depth, suggesting fluorescence by photopigments, or high light scattering paired with little absorbance by pigments at these bandwidths [36, 37].

### Photosynthesis and carbon fixation

**Fig. 4:**
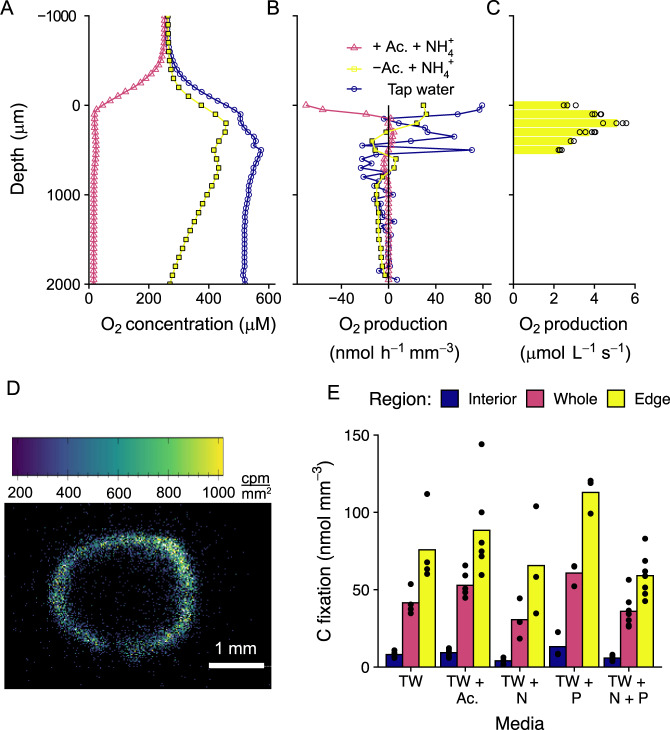
Photosynthesis. **A** Oxygen profiles were measured in the same photogranule under three different conditions: untreated tap water (blue circles), growth media without acetate but with ammonium (yellow squares), and growth media with acetate and with ammonium (pink triangles). **B** The total oxygen production at each depth of the photogranule, calculated from the profiles in figure A, assuming a spherical aggregate 4 mm in diameter. **C** Instantaneous oxygen production, determined by the change in oxygen concentration after 5–8 s dark shift, at six different depths, in media with ammonium but no acetate. The bars represent the mean oxygen production of 3–4 measurements, and the open circles are each individual measurement. **D** A representative microradiograph image of the distribution of ^14^C from carbon fixation in a photogranule (incubated with acetate). White scale bar is 1 mm. **E** Carbon fixation in photogranules incubated in the light in untreated tap water (TW), tap water amended with 3 mM acetate (TW + Ac.), with 8 mM NH_4_^+^ (TW + N), with 3 mM PO_4_^3−^, and with both 8 mM NH_4_^+^ and 3 mM PO_4_^3−^. Carbon fixation was measured by incubating photogranules with ^14^C–CO_2_ then determining ^14^C fixation with a microradiograph. The carbon fixation was segregated by region, using activity to mark boundaries within the aggregate: the interior of the photogranule (blue), the entire photogranule (purple) and the outer edge of the photogranule (yellow). The black dots represent measurements of individual photogranules. These light-exposed incubations were carried out simultaneously to incubations in the dark, which are displayed in (**A**). The results from incubations with 3 mM and 10 mM bicarbonate were combined, since there was no substantial difference between groups.

The photogranules had a high capacity for both oxygen production by photosynthesis and oxygen consumption (Fig. 4A–C). In the presence of acetate, oxygen consumption surpassed oxygen production, leading to anoxia throughout the majority of the photogranule, even in the light. In the absence of acetate, the photogranule was saturated with oxygen throughout (Fig. 4A) and had a maximum oxygen production of 738 nmol-O_2_ photogranule^−1^ h^−1^, integrating the oxygen production rate with depth (Eq. 1). Although the oxygen concentration was lower in the presence of acetate, photosynthesis did not decrease, resulting in carbon fixation comparable to in the absence of acetate (Fig. 4E). This indicates that oxygen is rapidly, nearly instantaneously cycled in the photogranule and that during periods of acetate exposure, respiration is oxygen-limited, despite high oxygen production through photosynthesis. The interior of the photogranule is also likely light-limited, as light is rapidly attenuated within the photogranule (Fig. 3), and thus the majority of the light-driven carbon fixation in the photogranule occurs in the outer edges (Fig. 4D, E). Nevertheless, the highest photosynthesis rates were ~200 µm below the surface of the granule (Fig. 4C), consistent with the microscope images showing a higher density of photosynthetic bacteria slightly below the surface of the granule (Figs. S3 and S4). Considering a photogranule with a diameter of 4 mm, a maximum carbon fixation rate of 383 nmol-C photogranule^−1^ h^−1^ was calculated (Eq. 1).

Carbon fixation in the photogranule was insensitive to short-term changes in nutrient concentration (Fig. 4D). Carbon fixation was similar in all photogranules incubated in untreated tap water, in tap water with 3 mM acetate, in tap water with 8 mM ammonium, in tap water with 3 mM phosphate, as well as in tap water with both 8 mM ammonium and 3 mM phosphate. This indicates that the rate of carbon fixation, driven by photosynthesis, is constant over the entire light phase of the reactor batch cycle. However, the rate of aerobic respiration and other oxygen-consuming processes, such as nitrification, varies substantially, due to the variable carbon supply.

### Nitrification/denitrification and carbon fixation

**Fig. 5:**
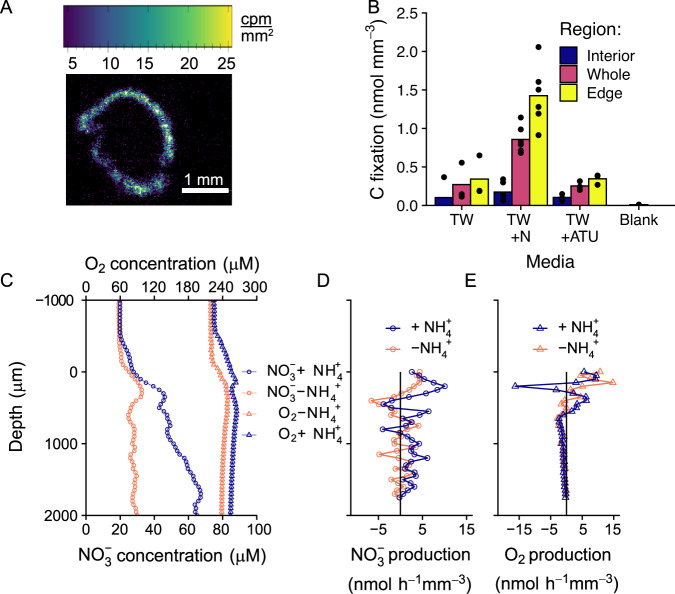
Nitrogen cycling. **A** representative microradiograph image of the distribution of ^14^C from carbon fixation in a photogranule incubated with 8 mM NH_4_^+^. **B** Carbon fixation in photogranules incubated in the dark in untreated tap water (TW), tap water amended with 8 mM NH_4_^+^ (TW + N), with 8 mM NH_4_^+^ and the nitrification inhibitor ATU (TW + ATU), and in photogranules killed with paraformaldehyde before tracer addition (Blank). Carbon fixation was measured by incubating photogranules with ^14^C-bicarbonate then determining ^14^C fixation by microradiography. The carbon fixation was segregated by region, using activity to mark boundaries within the aggregate: the interior of the photogranule (blue), the entire photogranule (orange) and the outer edge of the photogranule (yellow). **C** The average of three microprofiles of nitrate (circles) and oxygen (triangles) concentrations in the same photogranule incubated in the light first without NH_4_^+^ (orange) then with 100 µM NH_4_^+^ (blue) after reaching steady state. **D** Nitrate production at each depth calculated from the profiles in (**B**), in nmol h^−1^, assuming a spherical photogranule with a radius of 1800 µm. Positive values indicate zones of production, while negative values indicate zones of consumption. **E** Instantaneous oxygen production, determined by the change in oxygen concentration after 5–8 s dark shift, at six different depths, in tap water with ammonium. The bars represent the mean oxygen production of 3–4 measurements, and the open triangles are each individual measurement.

Autotrophic nitrification occurred largely in the outer edges (0–500 µm) of the photogranule in dark incubations with ^14^C-labelled carbonate, with or without ammonium and the nitrification inhibitor ATU (Fig. 5B). Carbon fixation was highest in the incubations containing ammonium (0.9 nmol mm^−3^) although there was still detectable carbon fixation in the incubations without ammonium and with ATU. The slightly higher level of carbon fixation (0.4 nmol mm^−3^) in photogranules incubated in untreated tap water compared to the photogranules incubated with ATU (0.3 nmol mm^−3^) suggests that the photogranules may have stored some residual ammonium or used the low concentrations of ammonium (<0.03 mg_NH4_ L^−1^ or <1.66 µmol L^−1^) available in the tap water (Table S1). The higher level of carbon fixation in photogranules incubated with ATU compared to dead photogranules (Blank, 0.06 nmol mm^−3^), indicates there is significant anaplerotic carbon fixation in the photogranule (0.2 nmol mm^−3^).

Profiles of nitrate concentrations in the light and dark partially confirm the distribution of nitrification predicted by the dark carbon-fixation distribution (Fig. 5B, C). While the absolute concentration of nitrate peaks in the photogranule centre (Fig. 5B), the volumetric rate of nitrate production, calculated from the same profiles, had has two peaks at the outer edge, and a relatively steady production in the centre. These peaks corresponded to a dip in oxygen concentration, and a peak in oxygen consumption, observed in multiple other photogranules as well (Fig. 5C–E). Nitrate consumption peaked in between the nitrification peaks, rather than at the photogranule centre. Note that diffusive flux through a sphere is dependent on the radius of the sphere (Eq. 1) which changes with depth. Thus, a linear gradient does not indicate that the system is controlled by diffusion, as it would in a flat biofilm. To aid interpretation of the profiles, reference gradients controlled by diffusive flux through a sphere and plane are plotted together (Fig. S5). Summing up the nitrate production rates at each depth an overall nitrate production rate of 93.7 nmol photogranule^−1^ h^−1^ (with NH_4_^+^) and 25.1 nmol photogranule^−1^ h^−1^ (without NH_4_^+^) was observed, which corresponded well with ^15^N-NH_4_^+^ incubations.

**Fig. 6:**
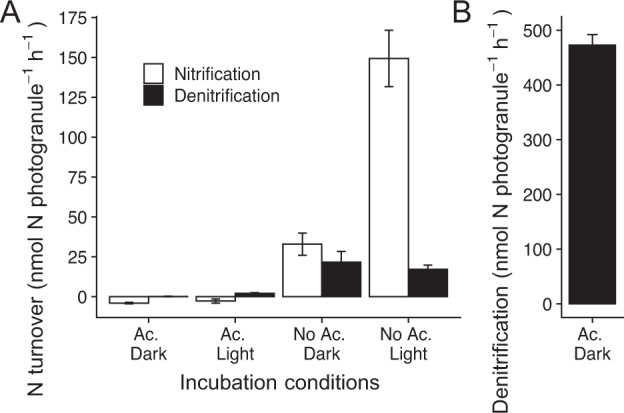
Rates of N-cycling processes. **A** Nitrification (white bars) and denitrification (black bars) in photogranules incubated with 1 mM ^15^N-labelled ammonium, either in the dark or the light, and with or without the addition of 3 mM acetate. Rates are a linear fit of the production of nitrate and nitrite or ^15^N–N_2_ in 4 separate vials stopped at different points. Nitrification represents rate of production of extracellular nitrate and nitrite plus the production of ^15^N–N_2_. Denitrification denotes the production of ^15^N-N_2_. Error bars indicate the standard error of the slope. **B** The rate of denitrification in photogranules incubated with ^15^N–NO_3_^−^ with acetate in the dark. The error bar represents the standard error of the slope.

Nitrogen cycling in the photogranules was either oxygen or carbon-limited, depending on the incubation conditions (Fig. 6A, B). In the presence of acetate there was no nitrification when photogranules were incubated with ^15^N-ammonium in the dark (Fig. 6A), due to an absence of oxygen. Some nitrification in the presence of acetate occurred in the light, as indicated by the production of ^15^N-N_2_ (black bar, Fig. 6A), although net nitrate was consumed from the background concentration of 10 µM (white bar, Fig. 6A). In the absence of acetate, nitrification was much higher, especially when incubated in the light (max. 150 nmol-N photogranule^−1^ h^−1^) (Fig. 6B). Denitrification was also higher in the absence of acetate, although the photogranules were always oxic in the absence of acetate, and there should be little free organic carbon (Fig. 6A). This indicates that there is significant carbon recycling within the photogranules, and that denitrification is tolerant to oxygen. Nonetheless, denitrification was clearly carbon-limited or depressed by oxygen in the absence of acetate, as indicated by the much higher rate of denitrification in photogranules incubated with ^15^N-nitrate in the dark, with acetate (max. 480 nmol-N photogranule^−1^ h^−1^) (Fig. 6B).

## Discussion

### Physical, biological, and structural features of photogranules

The stratification pattern in photogranules closely resembled that of photosynthetic microbial mats **(**Fig. 1). Motile filamentous cyanobacteria (e.g., *Alkalinema pantanalense*, *Leptolyngbya boryana*, *Cephalothrix komarekiana* and *Limnothrix* sp.) and excreted extracellular polymeric substances (EPS) generated a complex net of filaments that provided structural rigidity, similar to that observed in cyanobacterial mats [42, 43]. The proliferation of microbial communities within biofilms is dependent on the EPS matrix [44]. The lectin stain revealed that a large fraction of glycoconjugates in the EPS matrix was attributed to d-glucose and d-mannose. The glucose constituents were likely produced by filamentous cyanobacteria, as it is the dominant monosaccharide in the glycoconjugate fraction of their EPS matrix [23, 45, 46]. Both glucose- (especially α(1–4) glucans) and mannose-containing polysaccharides have been shown to play a key role in biofilm cohesion in both aerobic granules and phototrophic microbial mats [47, 48]. The lectin-specific BAN glycoconjugates reported show only a part of the total glycoconjugates present. Nevertheless, we anticipate that there are other types of glycoconjugates and matrix compounds present such as extracellular proteins and eDNA [49].

The importance of filamentous cyanobacteria in photogranule formation was highlighted previously [12, 19, 50]. It was proposed that initially, filamentous, and motile cyanobacteria form a nucleus of filaments (a bundle) that can harbour other organisms. As it grows this structure becomes more and more physically and biologically stratified and finally results in a photogranule, with a large diversity of microenvironments and associated microbial processes. In natural systems, as well as in photogranules, physical and biological stratification occurs according to the availability and gradients of light and nutrients [8]. Thus, while young and small photogranules (<0.5 mm) do not have a defined structure, photogranules grown to sizes of several millimetres show a clear stratification. Others have also observed microbial stratification in large (>2.5 mm) photogranules, with cyanobacteria forming a layer close to the surface [19]. In our study we found that independent of photogranule size, the filamentous cyanobacteria were present throughout the whole photogranule. However, they showed different arrangements of the filaments from surface to centre. While the cyanobacterial filaments were densely packed and jumbled at the surface and centre, in the area in between they aligned themselves radially. One explanation for this phenomenon is the competition for space within the photogranule, as radially arranging the filaments would facilitate movement between regions with higher exposure to light (phototaxis) and higher levels of other substrates (chemotaxis) [43]. This would be especially useful to “pierce” through the thick shell of non-phototrophic organisms in the first 500 µm of the photogranule, the region with the highest light availability. Such radial movement was previously observed in biogranules derived from subcultures of microbial mats composed of cyanobacteria (*Cephalotrix* sp. formerly known as *Phormidium* sp.) diatoms and heterotrophic bacteria originating from the North Sea [3]. Their movement was shown to be triggered by light and substrates.

### Functional stratification in photogranules

The photogranules also exhibited a functional stratification similar to phototrophic biofilms in nature. The photogranule surface was exposed to the highest light and substrate levels and supported the highest photosynthetic and nitrifying activities. In the presence of acetate, the photogranule harboured anoxic zones that allowed anaerobic processes such as denitrification to occur. Most of the microbial activity was concentrated in the outer 500 µm of the photogranule. In that region phototrophs (cyanobacteria and eukaryotic algae) attenuated about 90% of all incoming light and showed the highest photosynthetic activity at around 400–500 µm (max. 100 nmol/h).

In addition, to the high concentration of cyanobacteria in the outer part of the photogranule, there was also a large population of non-phototrophic organisms, including nitrifiers (*Nitrosomonas* sp., *Nitrobacter* sp. and *Nitrospira* sp.) and chemoheterotrophic bacteria/denitrifiers (*Thauera* sp. and *Zoogloea* sp.). This high microbial density supported high oxygen production/consumption and nitrate production, as well as carbon fixation (Figs. 4 and 5). However, the ^14^C incubations may underestimate the nitrification activity as they were performed in the dark (in the light carbon fixation by photosynthesis would be orders of magnitude higher than by nitrification, Figs. 4E, 5B) and nitrification was much higher in the light (Fig. 6). Nitrate profiles within the granules did indicate nitrification within the centre of the granule, although the highest rates were confined to the outer edge (peak ~200 µm) of the granule (Fig. 5D), which is slightly closer to the surface than in similar-sized cryoconites (peak ~400 µm) [5]. Similarly, the oxygen profiles in the absence of acetate but presence of ammonium also indicate some nitrification (i.e., oxygen consumption) in the centre of the granules, although again the highest rates were at the outer portion of the granule (Figs. 4B and 5D). Nevertheless, the nitrate accumulation observed in the profile (Fig. 5C) was not caused by increased nitrification rates at lower depth but mostly due to the reducing rate of nitrate consumption towards the centre. The peak in nitrification just below the surface also aligns with a model of photogranules grown without DOC, but the same level of ammonium as in this study. Given that DOC is rapidly consumed in the bulk liquid, of the scenarios modelled, this scenario most closely fits our data [21].

Although the centre of the photogranule seemed to be relatively inactive, it may serve important functions. These could include substrate storage (e.g., as lipids, starch or EPS), fermentative processes, or decomposition of dead organic constituents, which all contribute to internal nutrient cycling in the photogranule. The centre has a lower oxygen concentration and may shelter less oxygen-tolerant microbes. Anaerobic prokaryotes such as *Anaerolineaceae* and *Caldilineaceae* and the fungi *Trichosporon* sp. found in the photogranule can ferment carbohydrates and mineralize organic phosphorus and nitrogen, comprised in EPS or necrotic biomass, to H_2_, alcohols (e.g., butanol, ethanol), ketones (e.g., acetone), PO_4_^3−^, and NH_4_^−^, which in turn can be reused within the photogranule [51–53]. Cyanobacteria are also adapted to anoxic conditions and can ferment six-carbon sugars (e.g., glucose) to e.g., lactate or ethanol [54]. Such internal nutrient cycling likely increases in significance the larger the photogranule becomes and may fuel metabolic processes despite external substrate limitation.

### Nutrient removal and microbial activity during a sequencing batch cycle

The photosynthetic activity of the photogranule was insensitive to the short-term nutrient fluctuations typical of the wastewater batch process, although other microbial activities were substantially impacted. Combining our results with data collected from the wastewater reactor allowed us to build a timeline of the shifting nutrient limitations and metabolic processes that occurred over the course of a batch cycle (Fig. S9). At the beginning of a batch, nutrients such as ammonium (NH_4_^+^), phosphate (PO_4_^3−^), and acetate were available in high concentrations. In this phase, the photogranules could maintain high photosynthetic and heterotrophic activities, resulting in ammonium, phosphate, acetate, carbon dioxide, and oxygen consumption, and oxygen production. Only limited nitrification occurred during this phase, due to the low oxygen concentrations generated by aerobic respiration of acetate. Rapid growth of microorganisms during this phase fuelled ammonium and phosphorous uptake. As acetate concentrations decreased and oxygen concentrations increased, nitrification and subsequently denitrification kicked off. In the dark phase, the phototrophs switched from photosynthesis to respiration on internally stored photosynthates. Nitrification continued until all ammonium was converted to nitrate, even though nitrifiers made up only 2% of the total community. Since all acetate was already consumed, denitrification was fuelled by intracellularly stored carbon (e.g., as polyhydroxyalkanoates) [55] or internally recycled carbon by decomposition of organic matter [56] but was not able to completely remove all nitrate, due to carbon limitation.

The ^15^N incubations showed that denitrification can also occur under oxic conditions. Thus, denitrification was likely performed in part by the aerobic denitrifier *Thauera* sp. [57]. Further, the incubations showed that even in the absence of acetate the internally cycled organic carbon (e.g., organic carbon from phototrophs, or internally stored and recycled carbon) can sustain denitrification (Fig. 6A). Compared to the maximum denitrification rate with addition of acetate in the dark, the denitrification rate sustained on internally cycled carbon was about 25x lower (Fig. 6B). Nevertheless, these rates were sufficient to keep removing nitrogen from the bioreactor in the dark period.

### From ecology to application

The fundamental knowledge of how photogranules are structured and function will allow engineers to replicate this novel ecosystem for wastewater treatment. In our study we investigated an active phototrophic, nitrifying, and denitrifying community that exhibited high nitrogen and carbon removal/conversion rates and showed internally linked processes (exchange of oxygen, nitrogen, and carbon dioxide).

Introducing a phototrophic community to the wastewater treatment process will ultimately allow closure of the CO_2_ and oxygen cycles of the conventional activated sludge process. Oxygen would be produced through photosynthesis and CO_2_ by the heterotrophic conversion of organic matter, making an external supply of oxygen unnecessary. The oxygen production and consumption in the aggregate was calculated to be 13.6 mmol L^−1^ d^−1^ and 13.1 mmol L^−1^ d^−1^, respectively (Equation S1–S4), resulting in a net oxygen production. The oxygen produced by photosynthesis is quickly used by consumption processes, demonstrating closely coupled processes in the aggregate. In the beginning of the batch cycle when acetate was still present, the photogranule was oxygen limited, i.e., photosynthesis could not supply heterotrophic respiration and nitrification with sufficient oxygen to achieve maximal rates (Fig. 4A). This was also apparent from the hourly oxygen production rate of 1.1 mmol L^−1^ h^−1^ and oxygen consumption rate of 1.4 mmol L^−1^ h^−1^ in the first 4 h of the cycle (considering full removal of acetate after 4 h into the cycle and high nitrification activity). After acetate was fully consumed, photosynthesis would likely become carbon limited without external CO_2_ supply. To optimize oxygen demand versus oxygen production in a system without external oxygen or CO_2_ supply, the light supply and specific acetate load could be altered. This will be especially important when wastewater characteristics (N, P, COD) or light conditions change.

As stated before, most activity was in the edge (outer 500 µm) of the photogranule due to light penetration and nutrient diffusion limitation. The photogranules (2–4 mm) investigated in this study thereby contained a considerable relatively inactive zone, which reduced the overall conversion rate of an individual photogranule. Smaller sizes would optimize the conversion rate per individual photogranule and could consequently increase the maximal conversion rate of a photogranule treatment system. In previous studies, an ideal granule size was determined to be 1.25–1.5 mm for aerobic granules [58, 59] and 0.5–1.7 mm for photogranules [19]. While the light and carbon load of the system influence photogranule size, the solid retention time (SRT) ultimately controls the size of photogranules as it provides an upper ceiling to the age of a photogranule. A shorter SRT would result in smaller granules and higher photogranule-specific conversion rates but might be detrimental for some slow growing organisms (e.g. nitrifiers) and compromise settleability of the photogranule if granule sizes become too small (<0.5 mm) [60]. Therefore, granule size must be carefully evaluated with all these different aspects in mind.

Finally, our results indicate the need to change the operating conditions of the reactor to further increase nitrification rates. Nitrification was strongly controlled by the availability of oxygen, where rates were 3–4 times higher in the light than in the dark (Fig. 6A). In the presence of acetate, nitrification is expected to be completely inhibited, as the entire photogranule was anoxic under this condition. Nevertheless, during reactor operation, photogranules always completely removed ammonium from the supernatant, both when the batch started with a light cycle and when it started with a dark cycle. Since acetate consumption takes ~4 h (Fig. S8) the conditions for optimal nitrification would only be available for ~2 h in a cycle starting with a light phase. In contrast, in a cycle starting with a dark phase that consumes all acetate, nitrification would be optimized for a full 6 h. This indicates that there is substantial unused nitrification capacity in the cycle starting with a dark phase. An additional, partial exchange of the reactor liquid could be supported at this time, to increase reactor activity. This would also allow for the denitrification of more of the converted nitrate, which is otherwise carbon-limited, as most nitrate is produced after the removal of the acetate (Figs. 6B, S8). Another option would be to pulse an external carbon source (e.g., methanol, acetate, or molasses) to promote carbon-limited denitrification, which is a common practice in conventional wastewater treatment plants [61].

## Conclusions

Photogranules contain complete ecosystems in only a few cubic millimetres. A scaffold of motile filamentous cyanobacteria and EPS supports a diverse array of phototrophs and heterotrophs capable of nitrification, denitrification, photosynthesis, aerobic respiration, and phosphate uptake. This diverse community internally cycles at least three nutrients important in wastewater treatment: oxygen, nitrogen, and carbon. Oxygen from photosynthesis is immediately consumed by aerobic respiration and nitrification in the presence of acetate or ammonium, respectively. Nitrifiers convert ammonium to nitrate and nitrite, which is subsequently rapidly denitrified. Organic carbon fixed through photosynthesis is used to fuel denitrification after acetate is depleted. Conditions within the photogranule vary dramatically over time, from completely anoxic to oxygen concentrations three times above saturation, with even larger variation in nitrate and organic carbon availability. Nevertheless, photogranules maintain high, robust activity levels, supported by a dense network of interconnected cells that exchange nutrients. This dense network and nutrient exchange allow for denitrification in the absence of external organic carbon, small amounts of nitrification without the provision of external ammonium, and nitrification under nearly anoxic conditions. Photogranules thus offer an opportunity to utilize the sun’s energy to clean wastewater and decrease the energy requirement of conventional wastewater treatment, by reducing or eliminating the need to aerate treatment reactors.

## Supplementary information

Supplemental material

## Data Availability

The raw 16S and 18S rRNA gene sequence data is available in the EBI database under project number PRJEB54633. The other data that support the findings of this study are available from the corresponding author upon reasonable request.
