# Supplementary material for: High resolution functional analysis and community structure of photogranules
Source: ISME J. 2023 Mar 30;17(6):870–9. doi: 10.1038/s41396-023-01394-0 (PMC10203304; doi:10.1038/s41396-023-01394-0)
Supplement: Supplementary file 1 — Supplemental material [file 41396_2023_1394_MOESM1_ESM.pdf]

# High resolution functional analysis and community structure of photogranules

**Lukas M. Trebuch[1,2,†,*], Olivia M. Bourceau[3,†]**, Stijn M.F. Vaessen[1,2], Thomas R. Neu[4], Marcel Janssen[2], Dirk de Beer[3], Louise E.M. Vet[5], René H. Wijffels[2,6], Tânia V. Fernandes[1]

[1] *Department of Aquatic Ecology, Netherlands Institute of Ecology (NIOO-KNAW), Droevendaalsesteeg 10, 6708 PB Wageningen, The Netherlands*

[2] *Bioprocess Engineering, AlgaePARC Wageningen University, P.O. Box 16, 6700 AA Wageningen, The Netherlands*

[3] *Microsensor Research group, Max-Plank-Institute for Marine Microbiology, Celsiusstrasse 1, 28359 Bremen, Germany*

[4] *Microbiology of Interfaces, Department River Ecology, Helmholtz Centre for Environmental Research - UFZ, Brueckstrasse 3A, 39114, Magdeburg, Germany*

[5] *Department of Terrestrial Ecology, Netherlands Institute of Ecology (NIOO-KNAW), Droevendaalsesteeg 10, 6708 PB Wageningen, The Netherlands*

[6] *Faculty of Biosciences and Aquaculture, Nord University, N-8049, Bodø, Norway*

[†] *contributed equally*

[*] *Corresponding author:*
*Lukas M. Trebuch*
*Department of Aquatic Ecology, Netherlands Institute of Ecology (NIOO-KNAW)*
*Droevendaalsesteeg 10, 6708 PB Wageningen, The Netherlands*
*Tel: +31 (0)317 47 34 00*
*E-mail: L.Trebuch@nioo.knaw.nl*

# Supplemental material

## *Photogranules*

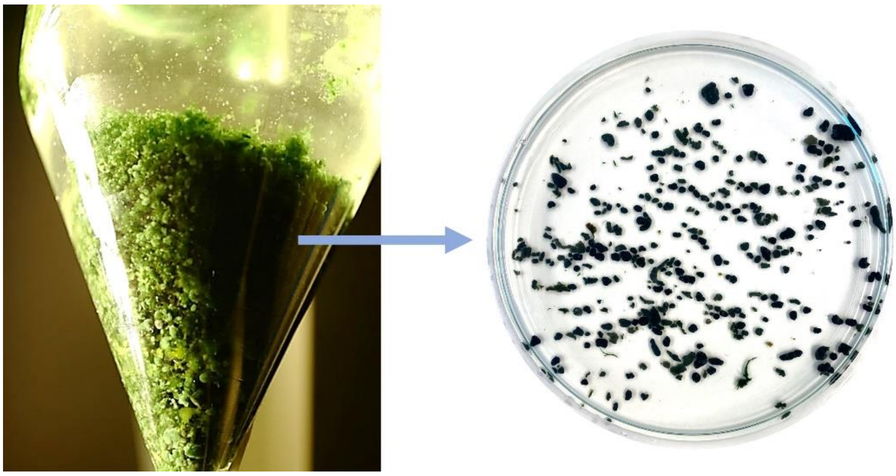

**Fig. S1:** Examples of photogranules that were used in this study: settled in the bubble column bioreactor (left) and in a Petri dish (right).

## *Microsensor measurements*

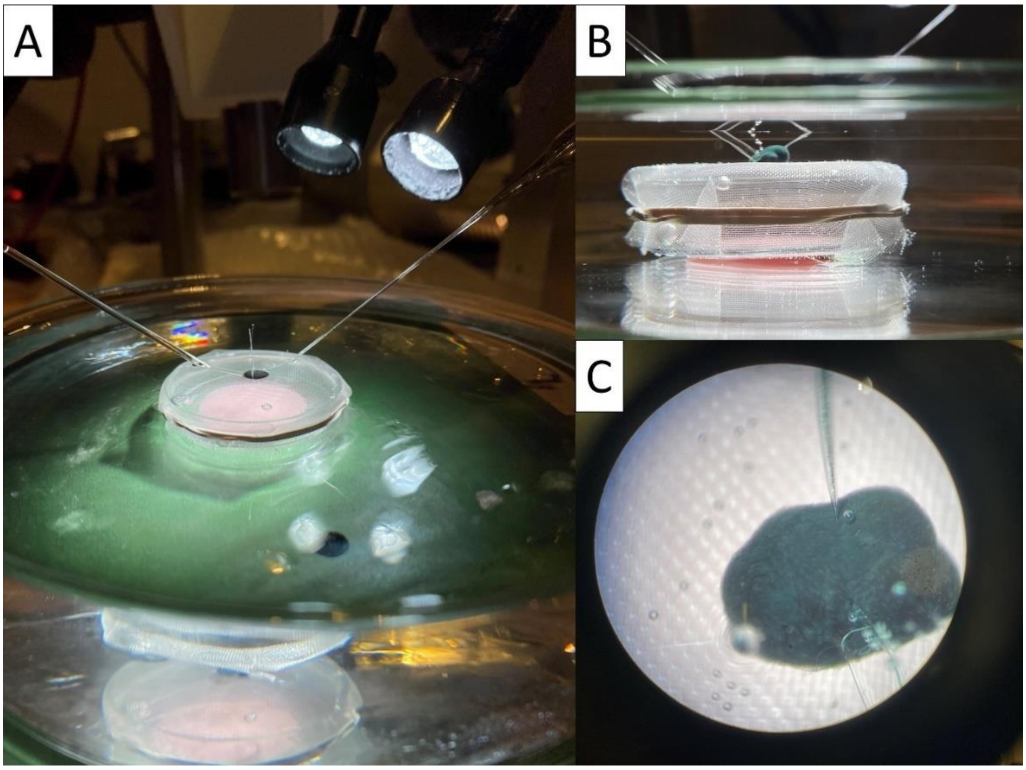


**Fig. S2:** Microsensor setup to investigate light, oxygen, and nitrate profiles in photogranules. The photogranule was placed on a nylon net suspended on a small glass Petri dish and pinned down by thin glass needles. The small Petri dish was placed into a larger glass Petri dish and submerged in liquid. Air was blown over the liquid surface by means of an air pump to mix the liquid. A halogen light source was used to illuminate the photogranule. (A) Image of the small Petri dish within the larger Petri dish. (B) Side view of the setup. (C) Image taken through a stereo microscope showing the microsensor penetrating the photogranule.

## *Tap water quality*

**Table S1:** Chemical analysis of the tap water of NIOO-KNAW used for experiments. The analysis was performed by the lab of the water supply company VITENS b.v.

| Parameter | Unit | Average | Minimum | Maximum | Numbers | Lower limit | Upper limit |
|---|---|---|---|---|---|---|---|
| Temperature in situ | *°C* | 11 | 10.5 | 11.6 | 14 | | 25 |
| Oxygen | *mg/L* | 7.2 | 6.9 | 7.6 | 14 | 2.0 | |
| Turbidity | *FTU* | 0.12 | <0.1 | 0.24 | 14 | | 1.0 |
| pH | *pH* | 7.92 | 7.82 | 8.04 | 14 | 7.00 | 9.50 |
| Saturation index | | -0.26 | -0.26 | -0.26 | 1 | -0.20 | |
| Conductivity at 20 °C | *mS/m* | 16.9 | 16 | 17.5 | 14 | | 125 |
| Carbon dioxide | *mg/L* | 2.1 | 1.6 | 2.7 | 14 | | |
| Aggresive carbon dioxide | *mg/L* | <1 | <1 | 1.4 | 14 | | |
| Hydrogencarbonate | *mg/L* | 92 | 91 | 94 | 14 | 60 | |
| Chloride | *mg/L* | 6 | 6 | 6 | 1 | | 150 |
| Sulfate | *mg SO4/L* | 8 | 8 | 8 | 1 | | 150 |
| Sodium (Na), after acidification | *mg/L* | 5.29 | 5.29 | 5.29 | 1 | | 150 |
| Potassium (K), after acidification | *mg/L* | 0.53 | 0.53 | 0.53 | 1 | | |
| Silicate | *mg Si/L* | 5.60 | 5.60 | 5.60 | 1 | | |
| Calcium (Ca), after acidification | *mg/L* | 29.5 | 28.8 | 30.4 | 14 | | |
| Magnesium (Mg), after acidification | *mg/L* | 2.2 | 2.14 | 2.32 | 14 | | |
| Total hardness | *mmol/L* | 0.83 | 0.81 | 0.85 | 14 | 1.00 | |
| Total hardness | *°D* | 4.6 | 4.5 | 4.8 | 14 | 5.6 | |
| Ammonium | *mg NH4/L* | <0.03 | <0.03 | <0.03 | 3 | | 0.20 |
| | *µmol/L* | <1.66 | <1.66 | <1.66 | 3 | | 11.09 |
| Nitrite | *mg NO2/L* | <0.01 | <0.01 | <0.01 | 3 | | 0.10 |
| | *µmol/L* | <0.22 | <0.22 | <0.22 | 3 | | 2.17 |
| Nitrate | *mg NO3/L* | <1.0 | <1.0 | <1.0 | 1 | | 50.0 |
| | *µmol/L* | <16.13 | <16.13 | <16.13 | 1 | | 806.40 |
| Ortho-phosphate | *mg PO4/L* | 0.06 | 0.06 | 0.06 | 1 | | |
| | *µmol/L* | 0.63 | 0.63 | 0.63 | 1 | | |
| Iron (Fe), after acidification | *mg/L* | 0.014 | 0.013 | 0.014 | 3 | | 0.200 |
| Manganese (Mn), after acidification | *mg/L* | <0.005 | <0.005 | <0.005 | 3 | | 0.050 |
| Aluminum (Al), after acidification | *µg/L* | <2 | <2 | <2 | 1 | | 30.0 |
| Antimony (Sb), after acidification | *µg/L* | <1 | <1 | <1 | 1 | | 5.0 |
| Arsenic (As), after acidification | *µg/L* | 2.77 | 2.77 | 2.77 | 1 | | 10.0 |
| Barium (Ba), after acidification | *µg/L* | 4.35 | 4.35 | 4.35 | 1 | | |
| Boron (B), after acidification | *µg/L* | <10.0 | <10.0 | <10.0 | 1 | | 500 |
| Cadmium (Cd), after acidification | *µg/L* | <0.10 | <0.10 | <0.10 | 1 | | 5 |
| Chromium (Cr), after acidification | *µg/L* | <0.5 | <0.5 | <0.5 | 1 | | 50 |
| Copper (Cu), after acidification | *µg/L* | 5.36 | 5.36 | 5.36 | 1 | | 2000 |
| Mercury (Hg), after acidification | *µg/L* | <0.02 | <0.02 | <0.02 | 1 | | 1 |
| Lead (Pb), after acidification | *µg/L* | <0.5 | <0.5 | <0.5 | 1 | | 10 |
| Nickle (Ni), after acidification | *µg/L* | <1.0 | <1.0 | <1.0 | 1 | | 20 |
| Selenium (Se), after acidification | *µg/L* | <0.5 | <0.5 | <0.5 | 1 | | 10 |
| Zinc (Zn), after acidification | *µg/L* | 4.77 | 4.77 | 4.77 | 1 | | 3000 |
| Cyanide, total | *µg/L* | <2 | <2 | <2 | 1 | | 50 |
| Fluoride | *mg/L* | 0.06 | 0.06 | 0.06 | 1 | | 1 |
| Colour intensity (455nm) | *mg Pt/Co/l* | <3 | <3 | <3 | 1 | | 20 |
| UV-extinction | *1/m* | 1.4 | 1.4 | 1.4 | 1 | | |
| Total organic carbon (TOC) | *mg/L* | <0.5 | <0.5 | <0.5 | 1 | | |

*Confocal laser scanning microscopy*

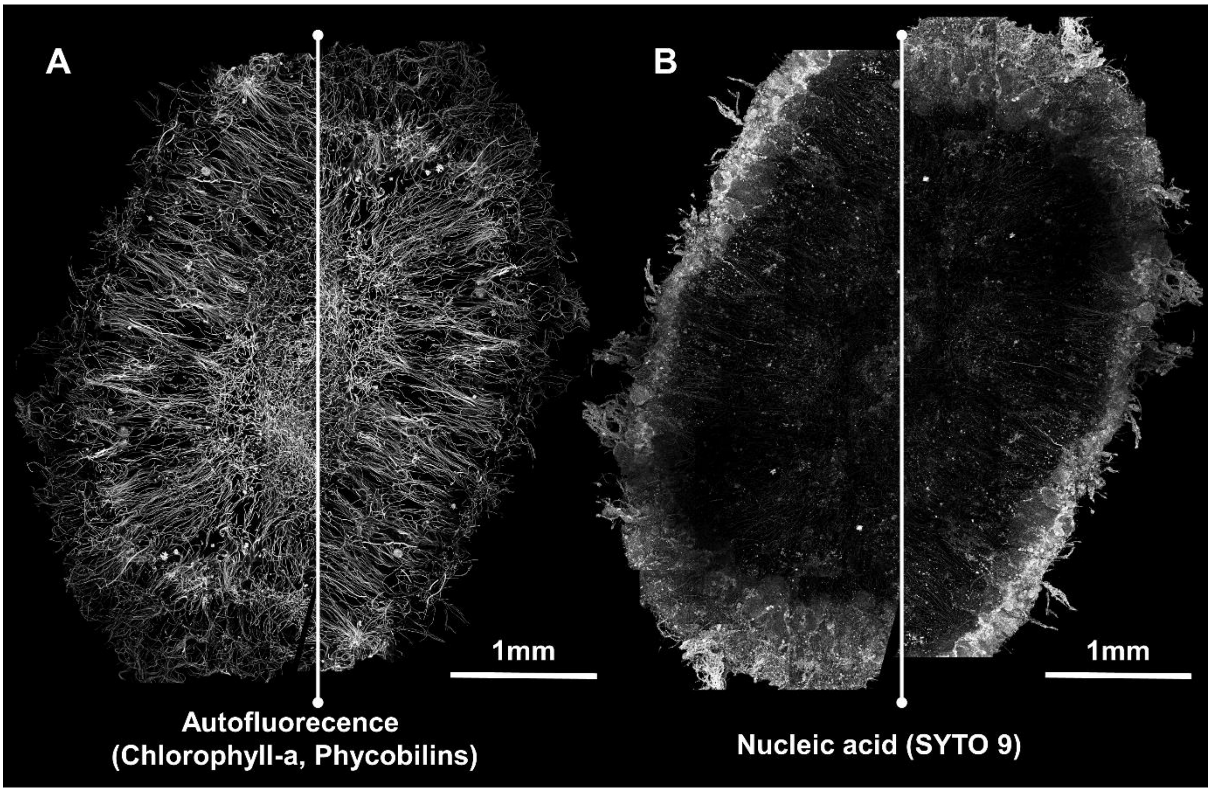


**Fig. S3:** CLSM image of a photogranule cross section showing: A) the autofluorescence signal of the photopigments chlorophyll-a and phycobilin and B) the nucleic acid signal (SYTO 9).

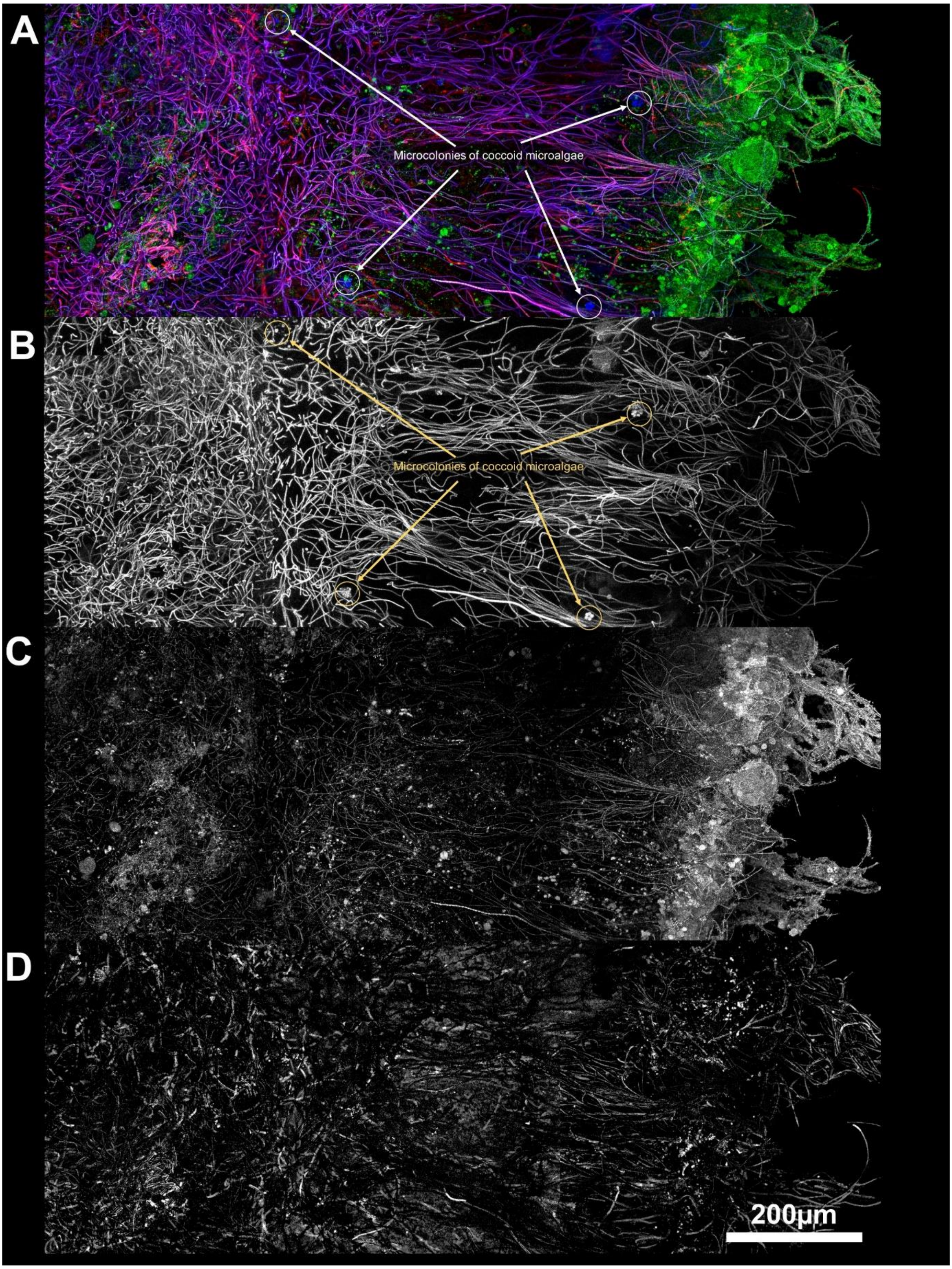


**Fig. S4:** Magnification of the cross section shown in figure 1C of the main text. This figure should highlight some structural features e.g., the glycoconjugates, which are difficult to make out in the zoomed-out version of the same cross section. **A)** CLSM image of a photogranule cross section showing nucleic acids (green), photopigments (blue, purple) and glycoconjugates (red). **B)** The same cross section only showing the blue channel which corresponds to chlorophyll-a. **C)** The same cross section only showing the green channel which corresponds to nucleic acids stained with SYBR green. **D)** The same cross section showing the blue channel subtracted from the red channel. This corresponds to the glycoconjugates stained with the BAN lectin. In panel A and B, microcolonies of coccoid microalgae are annotated.

*Microbial community composition*

*Please see table on the next page*

**Table S2:** ASV and taxonomy table of the 16S rRNA gene amplicon sequencing dataset. The 20 most abundant ASV are selected and presented in relative abundance with their respective taxonomic annotation. "NA" indicates that an ASV is no assign to a specific taxonomic level.

| ASV ID | Relative abundance | Kingdom | Phylum | Class | Order | Family | Genus | Species |
|---|---|---|---|---|---|---|---|---|
| ASV899 | 14.99% | Bacteria | Cyanobacteria | Cyanobacteriia | Leptolyngbyales | Leptolyngbyaceae | Leptolyngbya | NA |
| ASV765 | 13.28% | Bacteria | Cyanobacteria | Cyanobacteriia | Leptolyngbyales | Leptolyngbyaceae | Alkalinema | NA |
| ASV561 | 11.99% | Bacteria | Proteobacteria | Gammaproteobacteria | Burkholderiales | Rhodocyclaceae | Thauera | aminoaromatica |
| ASV113 | 5.75% | Bacteria | Planctomycetota | Phycisphaerae | Phycisphaerales | Phycisphaeraceae | SM1A02 | NA |
| ASV581 | 4.47% | Bacteria | Cyanobacteria | Cyanobacteriia | Cyanobacteriales | Phormidiaceae | Cephalothrix | komarekiana |
| ASV1261 | 4.39% | Bacteria | Proteobacteria | Alphaproteobacteria | Tistrellales | Geminicoccaceae | Candidatus_Alysiosphaera | NA |
| ASV504 | 3.78% | Bacteria | Armatimonadota | Fimbriimonadia | Fimbriimonadales | Fimbriimonadaceae | NA | NA |
| ASV1324 | 3.03% | Bacteria | Chloroflexi | Anaerolineae | Anaerolineales | Anaerolineaceae | NA | NA |
| ASV579 | 2.60% | Bacteria | Chloroflexi | Anaerolineae | Caldilineales | Caldilineaceae | NA | NA |
| ASV378 | 2.10% | Bacteria | Cyanobacteria | Cyanobacteriia | Limnotrichales | Limnotrichaceae | Limnothrix | NA |
| ASV1092 | 2.04% | Bacteria | Proteobacteria | Gammaproteobacteria | Burkholderiales | Rhodocyclaceae | Zoogloea | NA |
| ASV1048 | 2.00% | Bacteria | Planctomycetota | Phycisphaerae | Tepidisphaerales | Tepidisphaeraceae | Tepidisphaera | NA |
| ASV287 | 1.84% | Bacteria | Planctomycetota | Phycisphaerae | Tepidisphaerales | CPla-3_termite_group | NA | NA |
| ASV779 | 1.41% | Bacteria | Planctomycetota | Phycisphaerae | Phycisphaerales | Phycisphaeraceae | SM1A02 | NA |
| ASV1020 | 1.35% | Bacteria | Chloroflexi | Chloroflexia | Chloroflexales | Roseiflexaceae | NA | NA |
| ASV380 | 1.04% | Bacteria | Proteobacteria | Gammaproteobacteria | Legionellales | Legionellaceae | Legionella | dumoffii |
| ASV647 | 1.02% | Bacteria | Cyanobacteria | Vampirivibrionia | Obscuribacterales | Obscuribacteraceae | Candidatus_Obscuribacter | NA |
| ASV737 | 1.00% | Bacteria | Nitrospirota | Nitrospiria | Nitrospirales | Nitrospiraceae | Nitrospira | NA |
| ASV125 | 0.98% | Bacteria | Proteobacteria | Alphaproteobacteria | Rhizobiales | Xanthobacteraceae | Bradyrhizobium | NA |
| ASV889 | 0.87% | Bacteria | Proteobacteria | Gammaproteobacteria | Burkholderiales | Rhodocyclaceae | Zoogloea | resiniphila |
| **Phototroph** | **35.87%** | | | | | | | |
| **Denitrifiers** | **14.91%** | | | | | | | |
| **Nitrifiers** | **1.00%** | | | | | | | |
| **Anaerobs** | **5.64%** | | **Sum** | **79.96%** | | | | |

**Table S3:** ASV and taxonomy table of the 18S rRNA gene amplicon sequencing dataset. The 20 most abundant ASV are selected and presented in relative abundance with their respective taxonomic annotation. "NA" indicates that an ASV is no assign to a specific taxonomic level.

| ASV ID | Relative abundance | Kingdom | Phylum | Class | Order | Family | Genus |
|---|---|---|---|---|---|---|---|
| ASV1 | 33.86% | Eukaryota | Chlorophyta_ph | Trebouxiophyceae | Chlorellales | Chlorellales_fa | Chlorella |
| ASV3 | 18.15% | Eukaryota | Basidiomycota | Tremellomycetes | Trichosporonales | Trichosporonaceae | Trichosporon |
| ASV2 | 12.67% | Eukaryota | Chlorophyta_ph | Chlorophyceae | Chlamydomonadales | Chlamydomonadales_fa | Chlorococcum |
| ASV6 | 4.97% | Eukaryota | Chlorophyta_ph | Trebouxiophyceae | NA | NA | NA |
| ASV13 | 2.44% | Eukaryota | Chlorophyta_ph | Trebouxiophyceae | Chlorellales | Chlorellales_fa | Chlorella |
| ASV9 | 2.14% | Eukaryota | NA | NA | NA | NA | NA |
| ASV10 | 2.01% | Eukaryota | NA | NA | NA | NA | NA |
| ASV12 | 1.91% | Eukaryota | NA | NA | NA | NA | NA |
| ASV11 | 1.81% | Eukaryota | NA | NA | NA | NA | NA |
| ASV18 | 1.56% | Eukaryota | Cercozoa | Incertae_Sedis | Incertae_Sedis_or | Incertae_Sedis_fa | Gymnophrys |
| ASV14 | 1.53% | Eukaryota | NA | NA | NA | NA | NA |
| ASV17 | 1.50% | Eukaryota | NA | NA | NA | NA | NA |
| ASV16 | 1.49% | Eukaryota | NA | NA | NA | NA | NA |
| ASV21 | 1.19% | Eukaryota | Chlorophyta_ph | Chlorophyceae | Sphaeropleales | Sphaeropleales_fa | Botryosphaerella |
| ASV24 | 1.13% | Eukaryota | Chlorophyta_ph | Trebouxiophyceae | NA | NA | NA |
| ASV20 | 1.04% | Eukaryota | NA | NA | NA | NA | NA |
| ASV26 | 1.00% | Eukaryota | Chlorophyta_ph | Chlorophyceae | Sphaeropleales | Sphaeropleales_fa | Tetradesmus |
| ASV23 | 0.90% | Eukaryota | NA | NA | NA | NA | NA |
| ASV8 | 0.87% | Eukaryota | Tubulinea | Arcellinida | Echinamoebida | Echinamoebida_fa | Vermamoeba |
| ASV28 | 0.79% | Eukaryota | Chlorophyta_ph | Chlorophyceae | Chlamydomonadales | Chlamydomonadales_fa | Chlorococcum |
| **Algae** | **58.06%** | | | | | | |
| **Fungi** | **18.15%** | | | | | | |
| **Protist** | **2.44%** | | **Sum** | **92.97%** | | | |

*Calculations on carbon fixation rate*

The measured carbon fixation rates from the $^{14}C$ incubations were used to calculate the total carbon fixation rate of an entire photogranule ($R_{C-fix,photogranule,total}$) in nmol N/photogranule/h. The carbon fixation rate of the "whole" photogranule ($R_{C-fix,photogranule}$) given in nmol N/mm$^3$/h was multiplied by the volume of the photogranule (**equation S1**). In that particular the diameter of the photogranule investigated was 4 mm which resulted in a volume of 33.5 mm$^3$.

$$R_{C-fix,photogranule,total} = R_{C-fix,photogranule} \times V_{photogranule} \quad \text{equation S1}$$

## *Diffusion modelling*

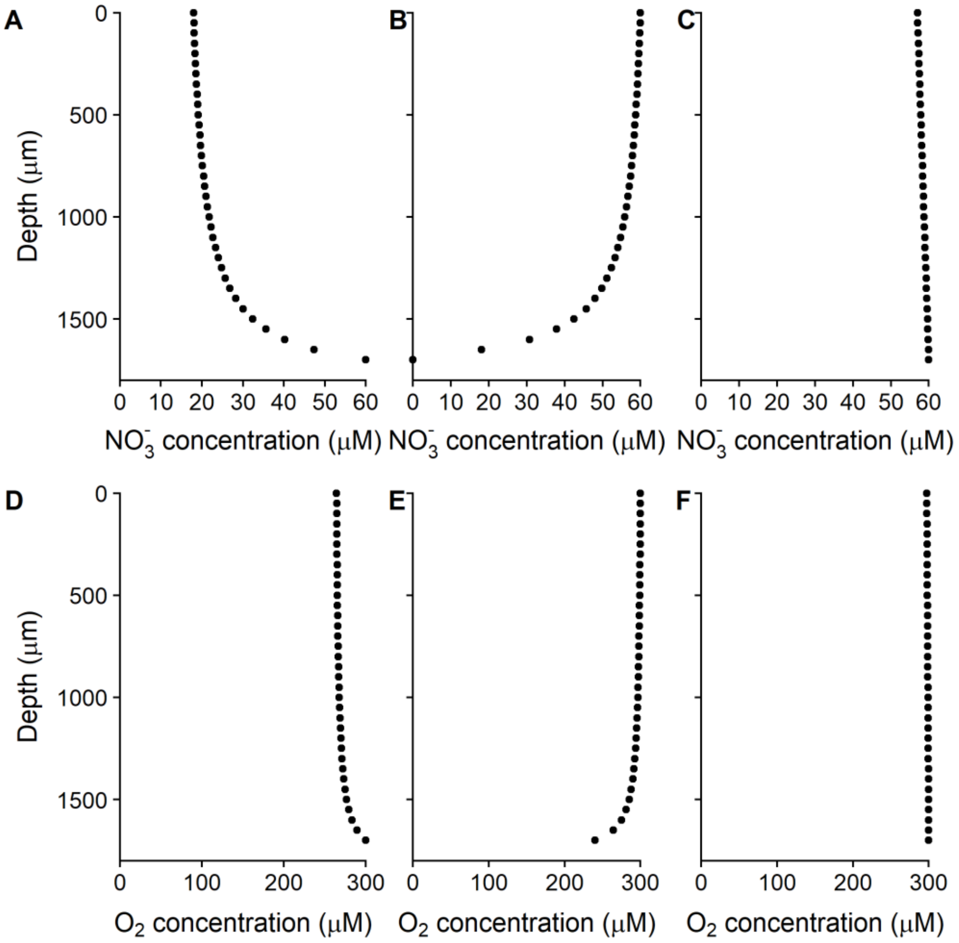


**Fig. S5:** Diffusion modelling: Diffusion gradients for nitrate (A-B) and Oxygen(D-E) through a sphere (A,B, D, E), or a plane (C,F) are plotted. A sphere with a radius of 1800 µm was assumed for the spherical plots, and a flux of $.7\times10^{-9}$ nmol $\mu m^{-2}$ $s^{-1}$ was used for plots A-C, and of $5\times10^{-9}$ for plots D-F nmol $\mu m^{-2}$ $s^{-1}$. Note that a diffusive gradient through a plane is linear, while through a sphere it is approaches a horizontal asymptote in the centre of the sphere, and a vertical asymptote towards the edge. This difference of the changing radius of the sphere with changing depth. The flux of a sphere is dependent on the radius, where J= dC/dx × Diffusion coefficient $\times 4\pi r^2$. If the flux is held constant, and the radius changes, dC, the change in concentration with changing radius, must also change.

## *Beta imaging analysis*

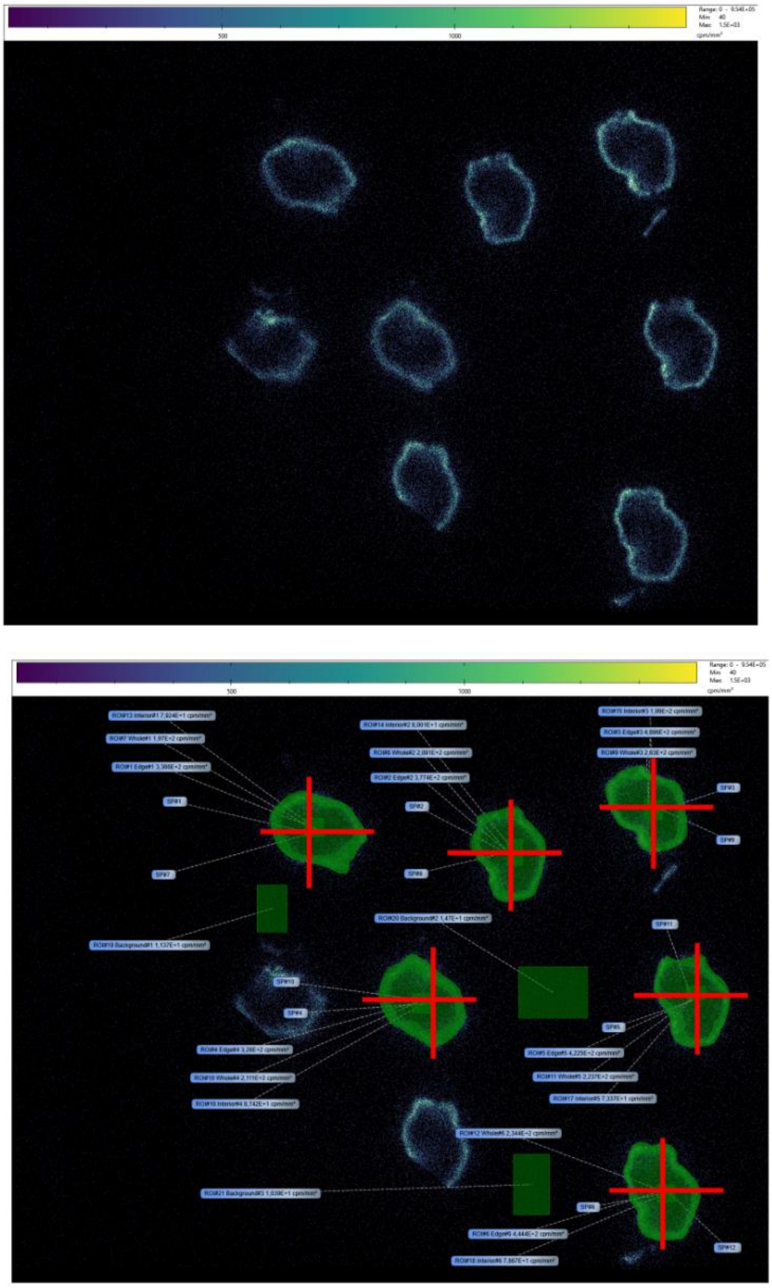

**Fig. S6:** Beta imaging analysis: The raw collected counts, per mm$^2$ of the photogranule slices from 1 representative slide (top) and the annotations (bottom). The high activity band was visually traced to collect the “edge” activity, then the whole granule was traced for the whole-granule activity. A small square in the centre of each granule was chosen for the “centre” activity. Squares in between granules were used to determine the background activity. Red horizontal and vertical lines are the paths for activity transects.

*Nutrient uptake during a 12h sequencing batch*

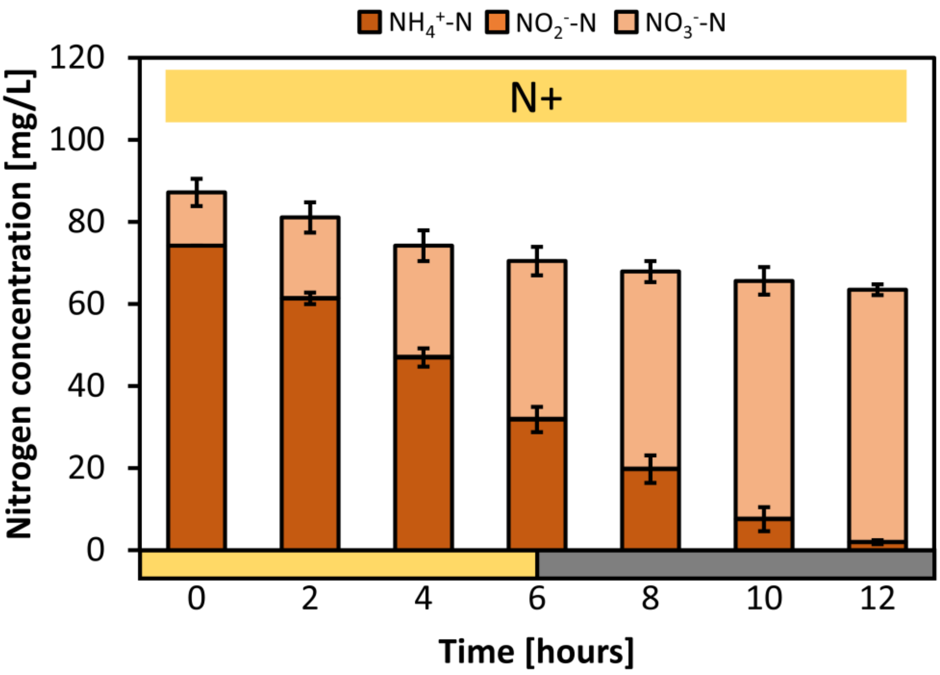


**Fig. S7:** Nitrogen evolution over the course of a sequencing batch cycle with 6 hours light followed by 6 hours darkness. The nitrogen species depicted are ammonium ($NH_4^+$), nitrite ($NO_2^-$) and nitrate ($NO_3^-$). The values presented are averaged over 5 timepoints over a period of 50 days. The error bars represent the standard deviation.

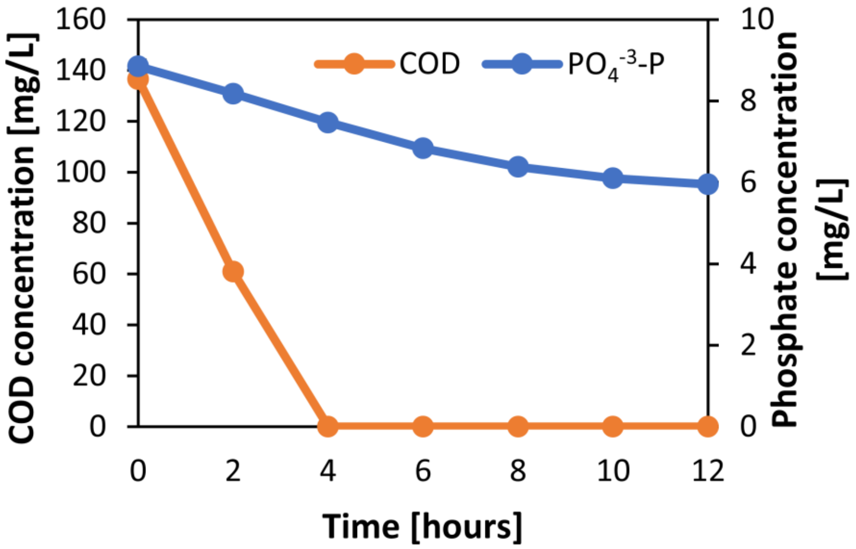


**Fig. S8:** COD and phosphate concentration over the course of a 12-hour sequencing batch cycle. The values presented are averaged over 5 timepoints over a period of 50 days. The error bars represent the standard deviation.

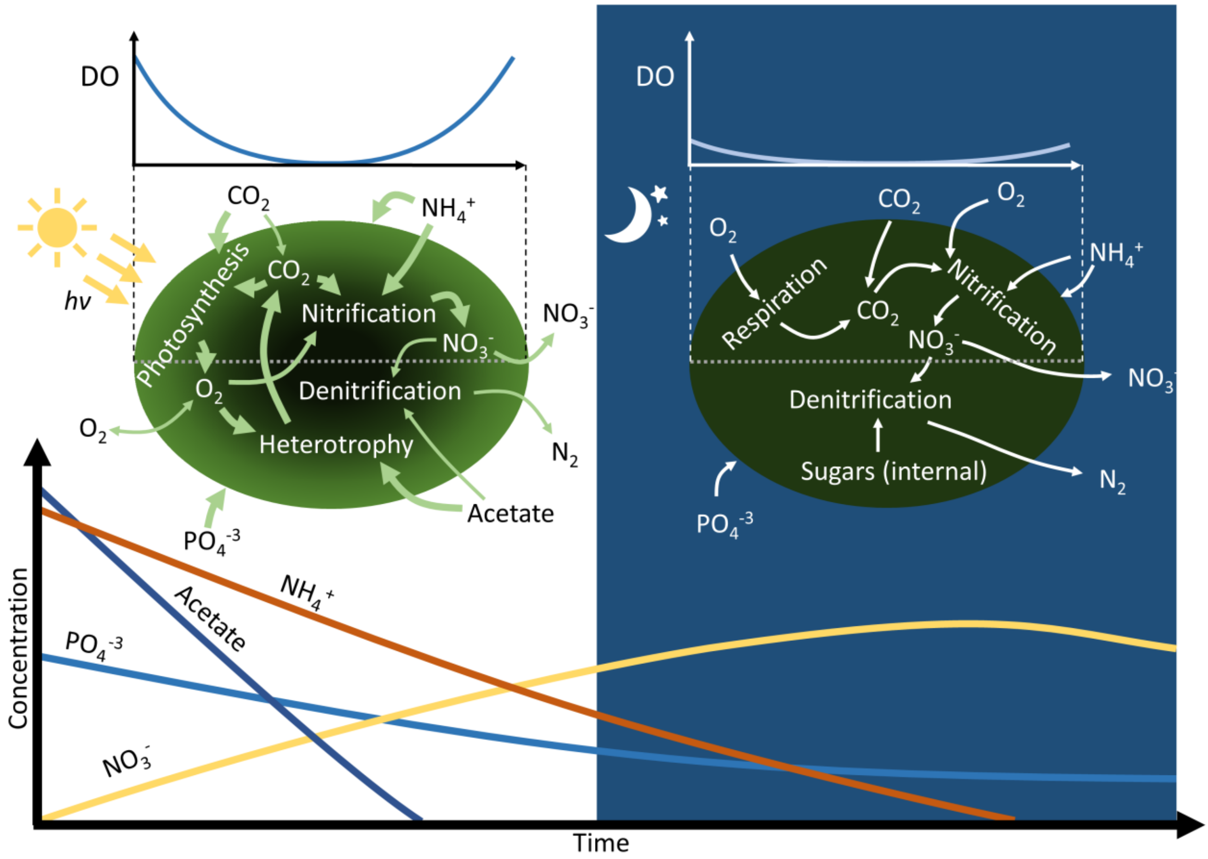


**Fig S9:** Schematic representation of the conditions and main metabolic processes occurring during a sequencing batch cycle in a photogranule. One cycle has 6 hours light followed by 6 hours darkness. On the left side the "light metabolism" of the photogranule characterized by photosynthesis, heterotrophy, nitrification and dentification is represented. On the right the "dark metabolism" of the photogranule is shown which is dominated by photorespiration, nitrification and to a lower extent with denitrification activities. The line thickness of the arrow indicates the magnitude of the conversion rate (the thicker the higher). As phosphate ($PO_4^{3-}$) is essential for all organisms, its consumption is attributed to biomass accumulation by the entirety of the photogranule. Ammonium ($NH_4^+$) is consumed by both nitrification (thinner arrow during light, thicker arrow in the dark) and biomass assimilation (thicker arrow during light, thinner arrow in the dark). The dissolved oxygen (DO) concentration over the cross-section of the photogranule is depicted on top of the two photogranules. This schematic representation is based on the microsensor measurements, $^{14}C$ and $^{15}N$ incubations and nutrient data from the bulk liquid in the bioreactor (figure S7 and S8).

*Calculating overall oxygen production and consumption*

The bioreactor received a total photon flux of 0.34 $mol_{ph}$/L/d ($I_{ph.V}$). This was calculated from averaged light measurements conducted over the entire reactor surface (0.528 $mol_{ph}/A_{reactor}$/d) divided over the reactor volume (1.6 L). When all incoming light is converted by photosynthesis to oxygen an oxygen production rate ($OPR$) of 13.61 mmol/L/d is obtained assuming photosynthesis to run at half of the maximal efficiency (0.04 mol-$O_2$/mol-photon) [1]:

$$OPR = I_{ph.V} \times 0.04 \qquad \text{Equation S1}$$

The oxygen uptake rate ($OUR$) is calculated according to the acetate load ($R_{HAc}$) and nitrification rate ($R_{NH4_{nitrification}}$) [2, 3]:

$$5CH_3COO^- + 1NH_4^+ + 5O_2 \rightarrow C_5H_7O_2N + 3H_2O + 5CO_2 + 5OH^- \qquad \text{Equation S2}$$

$$NH_4^+ + 0.0298NH_4^+ + 1.851O_2 + 0.1192CO_2 + 0.0298HCO_3^- \rightarrow 0.0298C_5H_7NO_2 + NO_3^- + 0.9702\,H_2O + H^+ \qquad \text{Equation S3}$$

$$OUR = R_{HAc} + R_{NH4_{nitrification}} \times 1.851 \qquad \text{Equation S4}$$

# References

1. Janssen M. Microalgal Photosynthesis and Growth in Mass Culture, 1st ed. *Advances in Chemical Engineering* . 2016. Elsevier Inc.

2. Liu G, Wang J. Probing the stoichiometry of the nitrification process using the respirometric approach. *Water Res* 2012; **46**: 5954–5962.

3. Boelee NC, Temmink H, Janssen M, Buisman CJN, Wijffels RH. Scenario Analysis of Nutrient Removal from Municipal Wastewater by Microalgal Biofilms. *Water* 2012; **4**: 460–473.
